# Staying with narrative: Stories of shame and gynaecological pain

**DOI:** 10.1353/lm.2023.a921569

**Published:** 2023-01-01

**Authors:** Katharine Cheston

**Affiliations:** Institute for Medical Humanities and Department of English Studies, Durham University, UK

**Keywords:** narrative, gynaecological pain, shame, healing, memoir

## Abstract

Storytelling is good for us – or so we are told. This article examines two memoirs, by Hilary Mantel and Susanna Kaysen, in which narrating experiences of gynaecological pain provokes shame and deepens pain. By attending to shame as a textual presence and asking what this shame can tell us about storytelling, I intervene in a long-standing debate about how to make sense of pain and illness, and whose responsibility this should be. Shame, I argue, reveals the presence of multiple (and often contrasting) illness *narratives*; I analyse these narratives, and their interplay, across Mantel’s and Kaysen’s memoirs. As scholarship moves beyond, past, or post-narrative, I urge us to stay: to interrogate the ways in which illness narratives interact – amplifying some stories and storytellers whilst fragmenting or silencing others – and to examine the responsibility we all have within this collective sense-making.

Whose responsibility is it to make sense of pain? Is narrating personal experience of illness always positive (healing, therapeutic) – or might the imperative to narrate afflict further pain? This paper interrogates these questions through an examination of two memoirs that detail experiences of gynaecological pain, and in which narrating this pain evidently provokes shame. By attending to shame as a textual presence and asking what this shame can tell us about illness narratives and our approach to them, this paper intervenes in a long debate about how to make sense of pain and illness. On one side of this debate are scholars (including Arthur Kleinman, Arthur Frank, Anne Hunsaker Hawkins) who have argued for the importance of expressing experiences of pain and illness in narrative; that coherent stories with a clearly-defined narrative arc have the power to restore order to, and create meaning from, what is otherwise a disordering, disempowering process.^1^ These first-person illness narratives (which might resemble a quest, or battle, or journey) are said to have therapeutic potential: to heal not only the person telling or writing the story, but also those reading or listening to it. More recent work has urged a critical reconsideration of such a narrative approach, encouraging us – as scholars of health and illness – to examine the limits of narrative, or even to look beyond it, to what might be *post-*narrative.^2^ For example, Sara Wasson’s analysis of accounts of chronic pain proposes reading ‘episodically’: attending to ‘the value of textual fragments, episodes and moments’ and hearing suffering ‘without imposing a narrative framework’.^3^ Moreover, Angela Woods’ sustained, vehement critique of narrative has centred around its ubiquity and presumed universality; she has called for us ‘to look again at what else lies in “our culture’s treasury of tool kits”‘ that might illuminate expressions of illness experience.^4^

This paper positions itself between these (often fraught) discussions and calls for a cease-fire. In attending to how shame fragments narrative – and renders storytelling painful, or impossible – I prompt readers not to look beyond, post- or past, but instead to stay (at least momentarily) with the concept of illness narrative.^5^ The term is typically understood to describe first-person accounts of illness as told by the ill person. Yet shame reveals the presence of multiple (and often contrasting) illness *narratives*; the wounded storyteller tells but one story amongst many others.^6^ Theirs is, of course, an important story – one to which we should listen closely, and not lose sight of – but it is one we must read amongst its kin: those (cultural, clinical) stories which make it possible (or not) and, perhaps, those stories which it precludes. In staying with narrative, I urge less of a critical reconsideration but rather a reorientation: a turn to look more broadly at all the ways in which we (as a society, as scholars, and as individuals) make storied sense of illness; at the ways in which these narratives interact (amplifying some stories and storytellers whilst silencing others); and at the responsibility we all have within this collective sense-making.

## Storytelling as healing?

Storytelling is good for us – or so we are told. The association of storytelling with healing has a long history. To note just one example, we might think of confession as it functions within the Christian tradition, in which sins are formed into (short) stories and recounted – whether privately in prayer, or to a priest in the confessional – so that spiritual healing, in the form of absolution, can occur. It is unsurprising, therefore, that telling stories of illness has been understood as a therapeutic process, at least in the Anglophone north. Howard Brody argues that ‘storytelling can itself be a healing activity’; that suffering is both ‘produced and alleviated by the meaning that one attaches to one’s experience’, and that the ‘primary human mechanism’ by which meaning is attached is through storytelling.^7^ Frank, too, believes ‘stories can heal’: stories are a central means of repairing, rediscovering or even reclaiming the self – damaged or wrecked by illness – which comes into being through stories.^8^ Moreover, storytelling is also understood to be good for those around us. Suffering needs stories, Frank tells us: in order to tell our own story of illness, we need others’ stories and, as such, the ‘wounded storyteller, ending silences, speaking truths, creating communities, becomes the wounded healer’.^9^ In this sense, storytelling is presented not only as a therapeutic possibility but also as a therapeutic *responsibility* – as a moral duty, an act that can heal both self and other. Empirical research attests to the healing powers of storytelling, which is built into practice in formalised narrative or writing therapies.^10^ Yet the rise in popularity of therapies such as EMDR (Eye Movement Desensitisation and Reprocessing), which explicitly eschew practices of reliving and recounting through narrative, could be said to undermine storytelling’s status as universal panacea.^11^ Is storytelling good for us, we might ask – or, if I may borrow from Woods’ intervention, in ‘focusing on the healing powers and potential of narrative’, have we overlooked ‘its capacity to harm and hinder’?^12^

With this in mind, I move to introduce the two memoirs through which I will interrogate this question, and those preceding it. Hilary Mantel’s *Giving Up the Ghost: A Memoir* (2003) and Susanna Kaysen’s The Camera My Mother Gave Me (2001) were not their authors’ first forays into published writing; Mantel had penned eight novels and would receive her first Man Booker prize within the decade, while Kaysen had two novels and a memoir (*Girl, Interrupted*) to her name.^13^ Although their cryptic titles give little away, both memoirs feature emotive accounts of gynaecological pain. Kaysen’s memoir offers a remarkably frank and unflinching account of her vaginal pain, which leaves her unable to wear trousers, drive her car, or have sex, without severe discomfort. Vividly figurative language, such as ‘my vagina felt as if somebody had put a cheese grater in it and scraped’ (*Camera*, 3), renders the severity of Kaysen’s pain undeniable – but its cause remains unclear. She receives an eclectic collection of diagnostic labels, and quizzes a wide variety of healthcare professionals, but still the reader is left wondering – as Kaysen herself later voiced in an interview – ‘[i]s it in the head or in the crotch?’^14^ Somatic or psychosomatic, that is the question. If Kaysen’s pain resists concrete diagnosis, Mantel’s resists correct diagnosis. Although references to vague ‘pains in my legs’ (*Ghost*, 147) are dotted throughout the first four parts of her memoir, it is only in the fifth, penultimate part that Mantel discusses her gynaecological condition in any depth – and the (mis)treatment she receives for it. When Mantel approaches her GP about her pain, she is sent to a psychiatrist who ‘soon diagnosed [her] problem: stress, caused by overambition’ (*Ghost*, 174); she is admitted to a clinic and prescribed psychotropic medication, with terrifying and traumatising consequences. Years later, Mantel investigates her own pain in the medical books of a university library; her suspected diagnosis of endometriosis is finally confirmed by a surgeon in London. Tragically, years of neglect have caused irreversible damage and, at the age of twenty-seven, her ‘reproductive apparatus’ (*Ghost*, 209) is surgically removed and she is left infertile.

Mantel and Kaysen tell the stories of their gynaecological pain, through their writing, to their readers – but this telling is also, in many ways, a *retelling*. The pages of their memoirs reverberate with dialogue, which these authors opt not to summarise or paraphrase but rather to recreate for their readers, who bear witness both to the narration of their pain as well as to the responses they receive from others. *The Camera My Mother Gave Me* is composed almost entirely of these reported, recreated conversations, which structure the memoir; chapters often end with Kaysen making another appointment, or being referred elsewhere, introducing the conversation readers will hear next. The reporting of these conversations is intriguing: Kaysen permits other voices (her boyfriend’s, her friends’, those of a variety of different healthcare professionals) to enter her autobiographical text unrestrained by speech marks, and this – combined with frequently absent dialogue tags – makes for a somewhat disconcerting reading experience, as the reader must work to pinpoint whose voice is whose. Mantel’s manner of recording conversations – with a variety of doctors and nurses, in hospital and in the community, in the UK and abroad – is similarly unusual. Unlike the frequent line breaks that punctuate Kaysen’s text, Mantel often opts to subsume her own speech, alongside (for example) a doctor’s, within the same paragraph, their words intermingling and unseparated.

Such authorial choices serve to draw yet more attention to these recreated conversations – and it is clear to their readers that narrating their experiences of gynaecological pain is not easy for either author. Telling their stories appears not therapeutic but painful, as both are seen to feel the sting of shame. In shame, we feel excruciatingly exposed: we might feel a desperate desire to hide, or feel frozen, incapable of movement; we might feel the blush rise in our cheeks; we might avert our gaze or bow our head.^15^ That shame can be painful needs, perhaps, no further elaboration – we can all, surely, recall an instance in our own lives that testifies to this connection – but still it sings from the literature in the metaphors appropriated for purposes of definition: shame is, for Andrew Morrison, ‘a sharp and searing feeling of failure and defectiveness about oneself’, while for Helen Lynd it is ‘a wound to one’s self-esteem, a painful feeling or sense of degradation’.^16^ It is a self-conscious emotion: when we feel ashamed, our attention turns inwards; the self judges the self and finds it to be wanting. Although these blushing, burning (yet fleeting) instances of acute shame are more easily brought to mind, shame can also become chronic: ‘not a discrete occurrence, but a perpetual attunement, the pervasive affective taste of a life’, as Sandra Lee Bartky so eloquently puts it.^17^ At times, Mantel and Kaysen refer to feelings of shame by name. For example, when Mantel describes explaining her surgery to her doctor in Botswana, she reflects: ‘I found it hard to talk; I thought I had nothing to be ashamed of, but somehow I felt ashamed’ (*Ghost*, 210). Most often, however, shame is revealed in altogether subtler ways. Although the reader cannot see Mantel and Kaysen blush or witness them avert their gaze, shame can be felt in their clear (and often explicit) discomfort at the autobiographical project – as they question themselves, and hide both information and themselves from their readers – as well as in the frequent gaps, and in all that is kept secret or silent. Much recent work has been dedicated to an analysis and theorisation of shame, focusing particularly on its chronic form and its connection with all aspects of medicine.^18^ (Indeed, I have contributed to this literature elsewhere.)^19^ For the purposes of this paper, however, I propose that it is enough to recognise its presence, to attend to the traces it leaves on the page, and to listen to what it can teach us about storytelling (and about the stories we tell).

That Mantel should ‘somehow’ feel ashamed, and find it ‘hard to talk’, is no surprise. Illness and pain have long been recognised as potentially shame-inducing experiences – but (if I may borrow Martha Nussbaum’s phrasing) some illnesses, and some pains, are clearly more marked out for shame than others.^20^ Mantel herself acknowledged this in an article she wrote for the *International Association for the Study of Pain*:

I have suffered from three painful conditions: gout, migraine, and endometriosis. […] Gout is, of course, recognisable in a straightforward way. It’s also, and I say this ruefully, largely a man’s disease. It trails some cultural baggage, and it involves some shame, but it doesn’t raise the same issues as those pains distinctive to women, which are to do with forbidden parts of the body.^21^

Indeed, gynaecological pain has been described in the scholarly literature as a ‘shameful pain’, precisely because it affects these ‘forbidden parts of the body’, and bodily functions (such as menstruation, sex), that are typically deemed improper topics for discussion – something to keep private, out of sight and speech.^22^ The fact that these are ‘pains distinctive to women’ can add an additional layer of shame. J. Brooks Bouson glosses the literature on femininity and shame when she writes that women, ‘conceived of as defective or deficient from male norms and as potentially diseased’, have ‘long been embodiments of shame’ in Western culture; even the healthy (cis)female body ‘remains a locus of shame for women, associated as it is with out-of-control passions and appetites and with something dirty and defiling.’^23^

Mantel’s shame is no surprise, but it is also no coincidence: shame is a natural response to the social world in which we find ourselves, and to the stories that circulate within this world. This, I believe, makes shame particularly well suited to an interrogation of narrative(s) and storytelling. Shame is, as Kaye Mitchell summarises, ‘both deeply personal and ineluctably social/relational’; it is felt painfully on the body, it shapes a life, but it necessarily occurs in the presence of the Other (whether this Other is real, imagined, or merely the internalisation of social mores).^24^ Of course, the scholarship on illness narrative has always been concerned with both personal and social/relational aspects of storytelling. ‘These embodied stories have two sides,’ writes Frank, ‘one personal and the other social’; the most obvious social aspect of these stories is that they are told *to* someone (whether that person is immediately present or not), while the ‘less evident social aspect of stories is that people do not make up their stories by themselves’. We draw the ‘plot lines, core metaphors, and rhetorical devices that structure the illness narrative’ from both personal and cultural models, Kleinman argues, while Frank claims that storytellers learn ‘formal structures of narrative, conventional metaphors and imagery, and standards of what is and is not appropriate to tell’ from their ‘families and friends, from the popular culture that surrounds them, and from the stories of other ill people’.

Shame prompts us to consider a different approach to this social aspect of storytelling. (I hesitate to describe this approach as novel – as Frank’s words, that ‘no-one can ever say anything new about stories or storytelling’, ring in my ears – but I do believe that it is urgent, and necessary, if potentially discomforting.)^25^ This approach is different in two ways. The first, which I have already touched upon, is that illness narratives are not limited to (what Kleinman terms) ‘personal narratives’: first-person accounts of the experience of illness, told by the ill person. The patient’s narrative has never been the only illness story. Other narratives abound: narratives, for example, in the clinical and scientific literature, that define an illness, determine who will be diagnosed with it and how (and by whom) they will be treated; or narratives that circulate in the media, or are shared in short snippets in a break room, shaping how clinicians and lay public alike view certain illnesses, certain diagnostic labels, and those who develop them. I detail how shame reveals this polyphony of narratives, as well as the (harmonious, discordant) interactions between them. Secondly, shame encourages us – just as I, in turn, encourage readers – to consider an alternative to existing models of narrative proliferation. Illness becomes, as Frank has it, ‘a *circulation of stories*’.^26^ Although Frank’s work has developed since publishing *The Wounded Storyteller*, his writing on narrative retains this optimistic tone: ‘one story necessarily leads to another’, in a flow of narratives which, we imagine, will continue indefinitely.^27^ Shame illuminates occasions in which this circulation of stories is interrupted: when, for example, an ill person wishes to speak of their experiences of illness – and perhaps even considers the language, the setting – but suddenly they feel ashamed, becoming silent and self-conscious, or hiding their face and fragmenting their own story. In what follows I interrogate this interruption – and ask what we can do about it.

This article follows this different approach through three distinct sections. The first explores Mantel’s and Kaysen’s narration of their gynaecological pain to their readers, showing how this narration can be painful, and potentially shame-inducing, for both authors and readers. Next, I turn to the clinical encounters depicted in both memoirs, examining Mantel’s and Kaysen’s narration of their pain (as recorded, and retold, to their readers) to a variety of clinicians; I explore the interplay of personal, cultural and clinical narratives, and the impacts this is seen to have on the writers. Finally, and to conclude, I propose answers to the two questions with which I opened this paper – and proffer additional questions as to what it might mean to stay with narrative.

## Storytelling through memoir

Mantel’s and Kaysen’s memoirs are neither diaries nor internal monologues: both authors appear acutely and immediately aware of their reading audience. Kaysen’s text opens with an untitled half-page of text – an unofficial prologue – in which she addresses the reader directly:

If you have a vagina you know that most of the time it is without sensation. How does your spleen feel? How do your kidneys feel? How does your pancreas feel? Luckily, we have no idea how these things feel. [*Camera*, 3]

Kaysen’s reader is not some disembodied presence but a real person, with a spleen, pancreas, kidneys – and, perhaps, a vagina. She interrogates this reader with three uncomfortably personal questions coming in quick succession, although the employment of the first-person plural (‘*we* have no idea’) adds a certain undercurrent of intimacy. Mantel, too, is conscious of her readers from the very first page of her memoir, when her stepfather’s ghost materialises: ‘I see a flickering on the staircase […] I know it is my stepfather’s ghost coming down. Or, to put it in a way acceptable to most people, I “know” it is my stepfather’s ghost.’ (*Ghost*, 1). Mantel’s self-conscious use of scare quotes, as well as her awareness of what might be ‘acceptable to most people’, suggests that she feels the ghostly presence of her readers just as keenly as she feels the presence of her stepfather’s ghost.

While the voices of Kaysen’s and Mantel’s readers may not literally interject in the body of their prose, these phantasmal readers – present yet invisible, seeing yet unseen – nonetheless wield significant influence: both authors appear vulnerable to experience shame under their imagined gaze.^28^ ‘Feeling that one is seen by a real or imaginary critical audience turns the self into an *object’*, writes Stephen Pattison: ‘The sense of being scrutinised from “outside” provokes a feeling of being objectified’, and this objectification is deeply shaming.^29^ Both authors are clearly aware that they are being seen and scrutinised – and take great pains to manage *how* they are seen. Storytelling is not, for these authors, an effortless, affectively-neutral process; their stories of pain do not flow, uncontrolled and unrestrained, from their bodies and onto the page. ‘I consider memoirs to be artifacts rather than spewage’, Kaysen declared in an interview, and Mantel appears to agree.^30^ Both authors have meticulously crafted the stories they tell their readers so as to simultaneously reveal and hide personal details; these are incomplete artefacts with gaping holes, in which every piece of information divulged only serves to accentuate those pieces still missing.

Privileging silence and secrecy over ‘spewage’, both Kaysen’s and Mantel’s memoirs are, I believe, perfect examples of the ‘“exhibited” kind of writerly shame’ that Denise Riley eloquently describes as ‘concealment tangled with unconcealment’.^31^ This entanglement is most visible in the means through which they disclose the gynaecological nature of their pain, their diagnoses, and other potentially relevant information. Mantel delays disclosing the full diagnostic picture of her pain. The first part of her memoir catalogues various ailments, including a fleeting mention of ‘bronchitis and lung inflammation’ (*Ghost*, 13) and a brief, yet articulate, allusion to prescription drug dependency: ‘I often began and ended each day with a sprinkling of barbiturates gulped from my palm’ (*Ghost*, 10). She writes especially eloquently of her ‘migraine headaches’ (*Ghost*, 2), her metaphorical flourishes – ‘a migrainous sleep […] plants on my forehead a clammy ogre’s kiss’ (*Ghost*, 4) – flying in the face of pain’s supposed inexpressibility.^32^ But Mantel also alludes to more mysterious pains – ‘there was a pain behind my diaphragm’ (*Ghost*, 12) and ‘a small ache behind my ribs’ (*Ghost*, 17) – which are echoed in the following three parts by repeated references to vague ‘pains in my legs’ (*Ghost*, 147).

The mystery of Mantel’s pains is only solved in the fifth, penultimate part of her memoir. Roughly half-way through this chapter – it is ‘Christmas week 1979’ – the clues suddenly become more obvious: Mantel is ‘in St George’s Hospital in London having [her] fertility confiscated and [her] insides rearranged’ (*Ghost*, 185). Still, it is only when she describes being examined by the ‘professor in charge of gynaecology’ (*Ghost*, 189) that the proverbial penny drops. In one clinical term (‘gynaecology’), the true nature of Mantel’s pain is revealed – but this revelation barely registers before Mantel proffers another, altogether more visceral. ‘When the professor had examined me at Outpatients,’ she writes, ‘I’d bled everywhere, on to his latex hands and the sheet beneath me’ (*Ghost*, 189). There follows a detailed description of endometriosis and its ‘dazzling variety of systemic effects’ (*Ghost*, 190), but it is this discomforting image of Mantel’s abject, bleeding vulnerability that is the most striking – and, undoubtedly, the most shocking, for readers who are (by this point) almost two-hundred pages into the memoir. Disclosing such personal information is evidently a source of great discomfort for Mantel, who pauses to reflect on the autobiographical project: ‘How can I write this, I wonder? I am a woman with a delicate mouth; I say nothing gross. I can write it, it seems, perhaps because I can pretend it is somebody else, bleeding on the table.’ (*Ghost*, 189-190). This short paragraph interrupts the story’s flow and lays Mantel’s shame bare on the page; the reader can almost feel her wincing as they hear her questioning herself. Yet the manner of this disclosure is also revelatory. Immediately after her the examination, Mantel tells her readers: ‘I sat in a chair: black vinyl, splayed legs, the ridge of its back hard against my spine’ (*Ghost*, 190). Following this grand, bloody unconcealment of the nature of her pain, she returns, once again, to concealment and euphemism; her delicate mouth can only hint, coyly, at the indignity of a gynaecological examination through her description of the chair with its ‘splayed legs’.

Unlike Mantel, Kaysen is immediately forthcoming, telling her readers in her unofficial prologue: ‘I have [a vagina], and something went wrong with it’ (*Camera*, 3). Kaysen addresses this topic in plain terms, with seemingly (and surprisingly) little embarrassment; she even specifies that the pain ‘occurred in one inch-long part on the left side. The rest of it was fine.’ (*Camera*, 3). Yet she, too, displays discomfort in disclosure, and similarly exhibits an entanglement of concealment and unconcealment that Riley associates with writerly shame. This is most apparent in her revelation about her boyfriend to the nurse at the alternative health clinic:

I felt at that moment that the alternative nurse and I were friends. Perhaps because of that, I said: I think he’s forcing me to have sex with him. […] He pesters me every night until I give him a blow job or let him fuck me. I do it so he’ll leave me alone. […] He holds my head down, I said. He holds me down by the back of my neck. I could barely bring myself to tell her this. […] Sometimes I feel that I’m choking, I said. [*Camera*, 102-103]

Despite feeling that she and the nurse ‘were friends’ – and the expletives and vulgar slang would certainly suggest a conversation between friends than between patient and professional – confessing these experiences appears to cause Kaysen great pain. She could ‘barely bring [herself] to tell her this’ and therefore reveals the violence of this sexual assault piecemeal, building detail upon distressing detail so as to progress from the euphemistic verb ‘pesters’ to the unequivocal admission: ‘Sometimes I feel that I’m choking’.

This is made all the more shocking by the fact that Kaysen waits until the mid-point of her memoir to announce that she has been sexually assaulted ‘every night’. Kaysen had many opportunities to alert her readership to her boyfriend’s abuse; she makes no secret of the difficulties her vulvar pain brought into their relationship, admitting in her second chapter, for example, that they ‘started having a lot of stupid arguments’ which ‘were really about [her] vagina’ (*Camera*, 13). Moreover, as Kaysen does not divulge her experience of sexual abuse directly to her readers – rather permitting them to *overhear* her confession to the nurse – any readers who may have mistaken Kaysen’s publication of intimate details about intimate parts of her body for an intimate, confessional story told to her readership will experience shock tinged with disappointment upon hearing this revelation. Disclosing, or *unconcealing*, her experience of sexual assault in this manner only serves to further emphasise Kaysen’s concealment. Both Kaysen and Mantel exhibit their shame as they send a clear message to her readers: you are neither seeing the full picture nor hearing the full story.

It is not just information, however, that Kaysen and Mantel keep from their readers. Both exert all their skill in writing to hide themselves from their readers – holding themselves at a distance, often obscured by and behind their prose. Kaysen remains an enigma throughout. Her voice is hidden amongst a polyphony of other voices, and she details more vivid descriptions of her vulva than of her general appearance; she disappears so frequently within her own memoir. Moreover, the reader knows about her vulvar pain, and (through the conversations she recreates) has witnessed her consult professionals, confide in friends, argue with her boyfriend. But all of these conversations relate to her pain; she discloses next to nothing about other aspects of her life. The time frame the memoir spans is kept vague: Kaysen tells her internist, just over half-way through the memoir, that she’s been ‘doing something about this [pain] for more than a year’ (*Camera*, 110), and in the final chapter she remarks to her gynaecologist that she has not seen him for ‘[a]bout a year’ (*Camera*, 155). Whether one or two years pass between the first and last page of the text is not clear, but either way it prompts the question: What else has happened during this time?

Mantel hides herself from her readers in an altogether more overt manner. In the first pages of her memoir, she reflects on the advice she would give ‘people who ask me how to get published’ and proposes the following imperatives: ‘Plain words on plain paper. Remember what Orwell says, that good prose is like a window-pane.’ (*Ghost*, 4-5). She then admits that she defies her own advice:

I stray away from the beaten path of plain words into the meadows of extravagant simile: angels, ogres, doughnut-shaped holes. And as for transparency – window-panes undressed are a sign of poverty, aren’t they? How about some nice net curtains, so I can look out but you can’t see in? How about shutters, or a chaste Roman blind? (*Ghost*, 4-5)

Plain words on plain paper are just too revealing: Mantel prefers the figurative cover offered by metaphorical meadows and ‘extravagant simile’. Rhetorical questions, too, collude in this concealment, disguising Mantel’s true intentions. Mantel’s metaphorical language overflows from this extract, but it is the visual metaphors that are the most fascinating – those ‘nice net curtains’, ‘shutters’, and ‘a chaste Roman blind’ that conjure up a whole array of coverings for Orwell’s window-pane. Mantel’s wish to write without being fully seen, to ‘look out’ without the reader seeing in, recalls the connection between shame and exposure. Pattison writes that ‘one of the main features of the experience of shame is a sense of *uncontrollable exposure*’, while Gershen Kaufman agrees that this sense of exposure is ‘inherent to shame’ and acknowledges its nefarious effects on the body, arguing that this shameful exposure ‘binds movement and speech, paralysing the self’.^33^ With masterful metaphors and figurative flourishes, both Mantel and Kaysen go to significant efforts in the hope of controlling this sense of uncontrollable exposure, shielding themselves from the ‘binding, almost paralysing […] effects of exposure’ that autobiographical writing leaves them vulnerable to experience.

The readers of these memoirs, however, might be more inclined to agree with Orwell. ‘Telling the truth’ is Paul Eakin’s first rule of autobiographical discourse, and readers certainly seem to expect the full truth, the whole truth, and nothing but the truth, from their autobiographical subjects.^34^ Mantel acknowledged post-publication that some people had ‘complained that there [were] big gaps’ in her memoir, while Kaysen admitted in an interview that, when it comes to writing memoirs, ‘[y]ou’re a bad sport if you don’t participate in total self-revelation’.^35^ Frank, of course, endows this total self-revelation with an ethical imperative, writing that ill people’s ‘storytelling is informed by a sense of responsibility to the common sense world and represents one way of living *for* the other’.^36^ Kaysen and Mantel resoundingly reject this ethical imperative and refuse to reveal all the gory details their readership so desires; their readers are left peering in at these window-panes of prose. But when the shutters are closed as these writers refuse, metaphorically-speaking, to bare all, their readers are equally vulnerable to shameful exposure. Silvan Tomkins argued that the ‘innate activator of shame is the incomplete reduction of interest or joy […] any barrier to further exploration which partially reduces interest or the smile of enjoyment will activate the lowering of the head and eyes in shame’.^37^ These memoirs are clearly replete with these ‘barriers to further exploration’, which could cause their readers to hang their heads in shame as their expectations of autobiographical writing are exposed to them. It is only when Kaysen and Mantel starve their readers of personal information that their readers are made aware of their greedy interest in every aspect of their autobiographical subjects’ lives. These authors’ refusal to fulfil their readers’ expectations for full disclosure and full exposure brings to light the cruelty of these expectations, which force writers of autobiographical texts to expose themselves to the paralysing, excruciating effects of shame for their readers’ pleasure. Neither author nor reader are safe from the pain of shame, evincing what Mitchell refers to as shame’s ‘contagious quality’: shame seeps out from the pages of these memoirs and ‘the reader cannot help but be affected/infected by it’.^38^ For Mantel, Kaysen, and their readers, telling stories about pain is a risky endeavour.

## Stories in the clinic

I turn now to the clinical encounter, to examine what sparks of shame are ignited as Kaysen and Mantel narrate their pain to a variety of healthcare professionals (and renarrate this, in their prose). Clinical encounters feature heavily in these texts; the voices of psychiatrists, gynaecologists and generalists alike speak authoritatively from their pages, often beginning chapters and sections, and often unrestrained by speech marks. It might seem strange that the voices of clinicians – imbued as they so evidently are with power and epistemic privilege – are permitted entrance into these autobiographical accounts of pain. Frank, for example, favours an approach that keeps ‘health-care workers […] in the background’. In his view, including the professional perspective – even if only to criticise it – detracts from the central aim of illness narrative: the subjective expression of illness experience, told in the patient’s own words. By including the practitioner’s perspective alongside their own (re)narration, Kaysen and Mantel detail a polyphony of intersecting narratives – and reveal their impacts. Focusing first on Mantel, and then on Kaysen, this section explores how some wounded storytellers – those, for example, who speak through female bodies suffering decidedly female pains – are more likely to be doubted and dismissed, and shows the sense of shame this causes to be excruciating.

Mantel’s childhood encounter with her family doctor sets the tone for the following conversations with clinicians that she includes in her memoir:

My arms and legs ache with a singing pain. The doctor says it is growing pains. One day I find I cannot breathe. The doctor says if I didn’t think about breathing I’d be able to do it. Frankly, he’s sick of being asked what’s wrong with me. He calls me Little Miss Neverwell. [*Ghost*, 82]

Four short sentences follow a repeated pattern, in which what the ‘doctor says’ has the power to contradict and negate Mantel’s suffering. In what could be perceived as a patronising parody of the diagnostic process, it is the doctor who is metaphorically ‘sick’, while the young Hilary’s literal sickness is dismissed, subsumed under the derisive moniker ‘Little Miss Neverwell’. Mantel’s pain, however, continues to resound; she writes in the fourth part that her ‘legs still ached with an old singing pain’ (*Ghost*, 147). These audible aches are echoed in Kaysen’s memoir, where readers hear her ‘vagina’s song of pain’ (*Camera*, 16) and onomatopoeic terms, such as her ‘zing of pain’ (*Camera*, 136), join in the chorus.

Yet when Mantel eventually approaches ‘the doctor, down at the Student Health Service’, he appears uninterested in her pain, enquiring instead about her marital status with a series of staccato imperatives that drown out Mantel’s singing aches: ‘Sick? […] Throw up? […] *Mrs?* You’ve got married? Pregnant, are you?’ (*Ghost*, 168). Little Miss Neverwell is now a grown woman, but still her pain is doubted and dismissed: Mantel recounts her appointment to her husband, ‘I said my legs ached and he said it was accounted for by no known disease.’ (*Ghost*, 169). Again, the doctor’s reported speech overrides Mantel’s, erasing her experience in one simple statement. Mantel’s voice – which expresses her experiences so eloquently and articulately in prose – is silenced in the clinical encounter; her attempted narration of her pain to her doctor is unsuccessful. Mantel tries again, and readers witness her attempts to (re)narrate her suffering, but this, too, is futile: ‘Go back, said my husband; tell them how you really are. Here you go, said the doctor, scribbling me a prescription; I think what you need is some anti-depressants.’ (*Ghost*, 171). In two sentences, Mantel summarises the story of her (attempted) storytelling: her voice disappears entirely from the clinical encounter, marked silently by the full-stop between imperatives spoken by Mantel’s husband and her male doctor.

The reasons for this disappearance soon become clear. When the prescribed anti-depressants prove ineffective, Mantel returns to the GP who ‘did what you do when someone says she is vomiting: send her to a psychiatrist.’ (*Ghost*, 173). Mantel’s employment of female pronouns is no accident; it is not her pain but her very *femininity* that is pathologised. The psychiatrist, Mantel writes, ‘soon diagnosed my problem: stress, caused by overambition’ and this, she stresses, ‘was a female complaint, one which people believed in, in those years, just as the Greeks believed that women were made ill by their wombs cutting loose and wandering about their bodies’ (*Ghost*, 174). Returning to (and reiterating) these beliefs pages later, Mantel states: ‘It was in the nature of educated young women, it was believed, to be hysterical, neurotic, difficult, and out of control’ (*Ghost*, 177). Mantel’s own, first-person illness narrative, told to her doctors, intermingles with other narratives – about women, their bodies, and the symptoms that afflict them. These narratives are culture- and context-dependent: that overambitious, educated young women were, by nature, neurotic was a story in which people believed, in the seventies – just as other cultures, in other centuries, had believed other stories, the residual (or, should I say, *hysterical*) traces of which still remain. These stories are pernicious, pervasive – but they are often unspoken, or even unconscious, as becomes evident when Mantel is admitted to the university (psychiatric) clinic and ‘[n]o one ventured a diagnosis: not out loud’ (*Ghost*, 177).

Still these stories sing out loudly – and amongst them, Mantel’s own story is silenced. Her frustration seeps from the page: ‘The more I said that I had a physical illness, the more they said I had a mental illness. The more I questioned the nature, the reality of the mental illness, the more I was found to be in denial, deluded’ (*Ghost*, 177). Moreover, in the clinic, her ‘speech turned into a symptom’ (*Ghost*, 177). On day leave, she goes shopping to buy a nightdress and, with her vision blurred by medication, she ‘misread the label, and came back with a size 16 instead of a size 10’ (*Ghost*, 179). She tells the nurses about this, ‘trying to lighten the tone’ – but alas, her nightdress is ‘viewed in a grave light’:

Why had I bought it? It was a mistake, I said, you see I… Didn’t you hold it up? they asked me. Well, no, I, I just liked the pattern, I… Didn’t you remember what size you were? Did you feel you didn’t know? Yes, I know my size, but you see, my eyesight, it’s misty, it’s because of the drugs I… oh, never mind. [*Ghost*, 179.]

The nurses’ pointed questions and Mantel’s pitiful replies are contained, intermingling, within the same paragraph, with neither line breaks to divide nor speech marks to separate. The power dynamics could not be more obvious; Mantel’s stuttering, spluttering incoherence marks a distinct change from her articulate prose. The ellipses, which always follow the autobiographical ‘I’, carve out a space for Mantel’s enforced silence, rendering it visible on the page. Mantel tries desperately to speak, to tell her own story of her own pain – but amidst cultural narratives of educated young women and their decidedly female complaints, her own illness narrative is dismissed, discounted.

What is so devastating about all of this is that Mantel was right. There is no sense of vindication in the author’s summary: ‘Those crippling spasms that had to be ignored, those deep aches with no name, those washes of nausea, were not evidence of a neurotic personality […] They were evidence of a pathological process’ (*Ghost*, 225-226). Mantel’s story provided this evidence, and its dismissal led to physical harm – including infertility alongside a severe reaction to psychotropic medication – upon which others have eloquently elaborated.^39^ The emotional harm is equally devastating. Decades on, the shame still burns brightly; Mantel admitted in an interview that when ‘I wrote my story, I re-experienced the shame of being disbelieved’.^40^ On the page, this shame is felt most painfully in Mantel’s evident self-blame. Shame and self-blame have a long and entangled history; Michael Lewis, for example, argues that a central, critical feature ‘in the elicitation of shame’ is ‘the issue of responsibility or self-blame’.^41^ Readers might expect Mantel to blame the doctors who silenced her story, and who told her to ignore her crippling spasms – but instead, she is seen to locate the blame within herself: ‘I wonder why, despite all, I did not insist, could not insist, that doctors paid attention to me and located my malaise.’ (*Ghost*, 226). Her repetition (‘I did not insist, could not insist’) directs the blame inwards – at the ways in which she told (or didn’t tell) her story, at her inability to make her doctors listen to her and act accordingly.

The pain provoked by this self-blame is all the more explicit in the following extract:

Like a cretin, like some dumb little angel, I had believed what I was told. I believed that the pains which ran through my body each month were part of the burden of womanhood. I didn’t say to my doctors, by the way, my menstrual periods are agony. I thought they would say, get away, you, little Miss Neverwell! And when I had, timidly, approached the topic, they’d said robustly, whoah, now, you don’t want to worry! Period pains? That’ll clear up, my dear, after you have your first baby. Just you wait and see! [*Ghost*, 209]

Mantel spits out insult after insult, culminating in the barbed, borderline-misogynistic ‘dumb little angel’. She had believed their stories – stories that told her that agonising menstrual pains were simply ‘part of the burden of womanhood’. She didn’t speak – or, when she did speak, she spoke timidly, whilst her doctors’ responses were robust, exclamative. It is not guilt that Mantel expresses but shame: in guilt the focus is typically on the act (here, Mantel’s silence) whereas in shame the act exposes something fundamentally bad about the self – or, to borrow Sara Ahmed’s apt summary, in shame ‘more than my action is at stake: *the badness of an action is transferred to me’*.^42^ This transference is revealed to be complete as a deluge of self-contempt flows through the following pages, including ‘I was old while I was young, I was an ape, I was a blot on the page, I was a nothing, zilch.’ (*Ghost*, 212). It makes for harrowing reading. That Mantel, after being so mistreated, should blame herself, feels like one of the memoir’s greatest tragedies.

I move now to Kaysen, to analyse her attempts at narrating her pain within the clinical encounters she includes in her memoir, and the responses she receives and records. Science is silent as to the cause of Kaysen’s vulvar pain – a fact she discovers shortly after her symptoms began. In the first chapter, ‘Gynaecology: Fungus’, Kaysen details the conversations she has with her gynaecologist across multiple consultations over three months. The chapter finishes with the question:

But what is it? I asked him. What’s wrong with me?

I don’t know, he said. Try the alternative health place. The mind and the body – he wiggled his hands around. You have no bacterial infection. You have no fungus. You have no herpes. You have no cancer. I can’t tell you why this is happening, but maybe they can. [*Camera*, 9]

Kaysen is told, in detail, all that is *not* causing her pain, but the only suggestion of an answer is the gynaecologist’s vague hand wiggling. That Kaysen receives no adequate explanation for her gynaecological pain is by no means uncommon: one study (published, incidentally, in the same year as Kaysen’s memoir) found that over half of those referred to gynaecology clinics were deemed to have an illness with no medical explanation.^43^

Thus begins a cycle of referrals. Kaysen consults many professionals who all make different diagnoses and recommend different treatments; she asks many questions about her pain, its causation, its treatment and its prognosis. Kaysen’s questions are, it seems, an attempt to form a coherent narrative – to understand why she is in pain, so that she might treat (or cure) it, as well as make meaning from it. These incessant questions, in turn, tell their own story – of Kaysen’s desperation to know what is happening to her, and of her (increasing) distress at not knowing. Unfortunately for Kaysen, not all clinicians are as forthcoming about the absence of explanation as her gynaecologist; her interrogatives are often ignored, or met with silence. Kaysen’s vulvologist, a man with ‘soft, mushy features’ whose ‘face resembled a vulva’ (*Camera*, 26) is a chronic offender. He often ignores her questions completely, for example when she asks, ‘Why does it hurt farther out?’ he responds, ‘Now we’re going to try the novocaine’ (*Camera*, 28). Even when he does utter a response, this is often non-verbal, a monotonous noise that only serves to emphasise his lack of explanation:

Why did this happen? I asked him.

Eh, he said. He shrugged.

What is it, anyhow?

Eh, he said.

[…]

I felt stymied. This operation, […] Is it comparable to the Bartholin’s cyst removal?

Um, he said. Uh.

I took that for a yes. [*Camera*, 29-32]

If this thoroughly one-sided conversation between Kaysen and her vulvologist – which, in full, spans seven pages – is frustrating for the reader, this is nothing compared to Kaysen’s evident irritation. Stymied, she is abandoned, answerless and adrift, hopelessly attempting to make sense of the vulvologist’s dissonant noises. Although the vulvologist does not explicitly doubt Kaysen’s pain, his demeanour is dismissive and disinterested, shown also through his body language as he ‘shrugged’. It is not only Kaysen’s questions that are ignored: her deep emotional pain and her desperate need to know *why* this has happened to her are also brushed aside.

This experience is intensely distressing for Kaysen, who admits to her internist that ‘the whole visit’ to the vulvologist ‘was very upsetting’ (*Camera*, 39) and declares vehemently to her gynaecologist ‘I hated this man’ (*Camera*, 41). However, what seems most upsetting for Kaysen is that other explanations for her pain are permitted to flourish in this gap left by medical and scientific knowledge. Kaysen’s boyfriend, for example, stubbornly blames Kaysen herself for her vulvar pain. He is confrontational: ‘They think it’s all in your head, right? […] They think it’s all in your head, he said again.’ (*Camera*, 66). ‘They’ refers to Kaysen’s doctors: her boyfriend clearly puts great trust in what the doctors ‘think’, and when their verdict is not forthcoming, he declares Kaysen guilty. He is infuriated by the changes Kaysen’s pain has brought into their relationship, and he takes his anger out on Kaysen herself:

We never fuck anymore, he said.

I can’t!

You don’t want to, he said. [*Camera*, 99]

Deliberately confusing will with ability, Kaysen’s boyfriend creates a story in which her behaviour is an active choice, rather than a side-effect of her pain. When Kaysen circulates this story – of her boyfriend and his response – to her friends, they, in turn, create their own narratives from it. For example, her friend Paula blames Kaysen’s pain on her relationship with her boyfriend, voicing a concern the reader may share: ‘But really, don’t you think some of it has to do with the relationship?’ Kaysen replies, stutteringly, in the affirmative: ‘Yes, I said. And that gets me more worried. If I made it all up.’ (*Camera*, 79).

As others clamour to fill the silence left by science – and narratives abound – the seeds of self-doubt are sown. Kaysen’s concerns that she may have ‘made it all up’ ripple throughout her memoir. When she experiences dramatic side-effects from her prescribed pain medication, her immediate reaction is to doubt her own perception of her body’s sensations: ‘I wondered if I could be making it up.’ (*Camera*, 74). She frequently voices these concerns to friends and kindly professionals, for example asking the nurse at the alternative health clinic, ‘Is this some way of turning against [my boyfriend]? […] Is this a hysterical illness?’ (*Camera*, 63). Unlike her vulvologist and gynaecologist, this nurse gives a direct answer – a simple ‘No’ – and, moreover, she appears to hear the deep-seated worries that underlie Kaysen’s questions, as she expands, gently, upon this: ‘This is part of what’s so bad about this disease. People feel responsible for it.’ (*Camera*, 63). Later, with little progress and (still) no answers, Kaysen returns for another appointment with this nurse, who now suggests they examine a different path in the search for explanations:

Maybe the psychological issues – she began.

But then I feel responsible! I started to cry. I feel it’s hysterical. I feel that anyhow. [*Camera*, 101]

Kaysen interrupts the nurse and – for the first and only time in the memoir – she breaks down and sobs. The ripples of self-doubt crash into a wave of self-blame which, as for Mantel, is clearly associated with a painful sense of shame. Unlike Mantel, Kaysen is not directly confronted with (cultural, clinical) narratives of neurotic, hysterical women; she is not given a diagnosis (of sorts) of ‘stress, caused by overambition’; none of the clinicians she consults utter the word *hysteria*, nor do they employ any of its storylines. Yet, still, these stories seep in and, with no better explanation, Kaysen feels her illness is hysterical. Alongside Mantel’s tragic declarations of self-blame, this image of Kaysen – despondent, despairing, in tears – lays bare the affective textures of narrating experiences of gynaecological pain, from which we should not look away.

## Conclusion

To conclude, I return to the two questions with which I opened this paper: Whose responsibility is it to make sense of pain? Is narrating personal experience of illness always positive (healing, therapeutic) – or might the imperative to narrate afflict further pain? I shall begin with the latter. My analysis shown that, for Kaysen and Mantel, narrating experiences of pain (both in person, to their clinicians; and to their readers, through their writing) can lead to shame and thus further pain. They hide from their readers, obscuring their faces, their facts, and their voices in fragmented, cryptic texts that are replete with gaps. Some might argue that this reading is presumptive; that this additional pain may, ultimately, have been worthwhile and that this storytelling process was a healing one. But Mantel’s and Kaysen’s comments, post-publication, support my reading. Mantel, of course, remarked in an interview that, when she wrote her memoir, she ‘re-experienced the shame of being disbelieved’.^44^ The author did not expand on this comment; this particular shame was lived in private, and we can but imagine the pain it might have entailed. Kaysen is altogether more vehement on this topic, and her words have such a conclusive finality to them that I have saved them, so to speak, for last. When asked in an interview whether *The Camera My Mother Gave Me* ‘was therapeutic to write’, she did not hold back: ‘Not exactly. I don’t like that notion that writing is therapeutic. Therapy is therapeutic. Writing is writing.’^45^ Of course, this does not preclude the fact that storytelling can, for some, be a therapeutic process. I do not deny this, but instead offer an intersectional perspective, and call for more nuance – for a *but*. Storytelling can be good for us, but for some it can cause further pain; it can (as Woods puts it) ‘harm and hinder’, and it can (in a way that is both proverbial and literal) add insult to injury.^46^

I now turn to the first question – Whose responsibility is it to make sense of pain? – and on this topic there is, I believe, much we can learn from recent developments within the scholarship on shame. It used to be widely acknowledged that, as Bouson has it, the ‘way out of shame’ was by transforming shame into story: through ‘the recognition of shame and the narration of the shame story’.^47^ In order to rid ourselves of shame, these arguments hold, we must narrate it before others in order to purge and thus cleanse ourselves of it. (Of course, this harks back to the example of confession, which I included in the introduction.) More recently, however, scholars such as Mitchell have resisted such arguments of catharsis and confession and have instead posited shame as pervasive, structural: something that cannot be overcome by individual efforts.^48^ Shame is social – it is alive in societies, cultures, relationships – and, as such, the responsibility to find the ‘way out of shame’ should be collective, as opposed to being borne solely by the individual experiencing it.^49^ Might the same hold true for pain and illness: that the way to make meaning from these experiences – to make storied sense of them – might not be the sole responsibility of the ill individual, but a collective responsibility, carried by us all?

On some level, it is comforting to think that telling stories of illness is therapeutic: to believe that the ill person can regain control of their lives, heal themselves (and those around them), find meaning in what had seemed meaningless, and rediscover a sense of empowerment at a time when (we might imagine) they felt at their most powerless. This, in itself, is a story. Indeed, it is a reassuring one, because it tells us that even if the worst happens, there’s always something we can do: we can tell our story, and others will hear it, and they will help us. But we know – from reading Kaysen’s and Mantel’s memoirs, amongst so many other stories – that, all too often, this isn’t the case. Rather than imploring them to speak louder (even though this might cause them additional pain), it is time to consider *why*. This article has revealed the presence of multiple illness stories, including cultural and clinical narratives – such as those related to women, their pain, and their (mis)interpretation of it – that shame women with gynaecological pain, like Kaysen and Mantel, into silence. It should not be, I argue, the sole responsibility of the ill person to make meaning of their pain through storytelling (although, of course, routes to and spaces for such storytelling should be kept open for those who do want to speak or write their experiences). Instead, we need to look at how we are all, collectively, making storied sense of pain and illness – and, crucially, we need to expose and challenge the shaming and silencing narratives that circulate within our communities and healthcare systems. We need, too, to call for structural change so that we might be able to tell different stories; four decades after Mantel’s disastrous diagnostic debacle, it still takes (on average) the best part of a decade for a woman in the Western world to receive a diagnosis of endometriosis, while women (like Kaysen) still regularly find that their pain is unexplained.^50^

I finish this paper with a provocation – one that is far less comforting than stories of therapeutic storytelling. I speak, now, particularly to scholars within the medical humanities: a field still, as Woods puts it, ‘under the thrall of narrative’. Why has the focus on narrative within our interdisciplinary field been so one-sided – so focused on the individual, to the exclusion of the collective, the structural, and the social? Might this exclusive focus have protected us from having to consider our own part in the creation, promotion and promulgation of illness narratives? As scholarship moves beyond narrative, I urge us to stay, and to ask ourselves: what narratives are we telling, through our work? Whose stories might we be silencing?

## Endnotes

1 Frank, *The Wounded Storyteller*; Kleinman, *The Illness Narratives*; Hawkins, *Reconstructing Illness*.

2 Woods, ‘The Limits of Narrative’; Woods, ‘Beyond the Wounded Storyteller’; Woods, ‘Post-Narrative — An Appeal’.

3 Wasson, ‘Before Narrative’, 106, 111.

4 Woods, ‘Post-Narrative — An Appeal’, 404; Woods, ‘The Limits of Narrative’; Woods, ‘Beyond the Wounded Storyteller’; Woods, Hart, and Spandler, ‘The Recovery Narrative’.

5 For a contrasting yet complementary account of what it might mean to stay with narrative – and, in so doing, to consider the multiplicity of illness narratives – see: Bolaki, *Illness as Many Narratives*.

6 Frank, *The Wounded Storyteller*.

7 Brody, *Stories of Sickness*, 12–13.

8 Frank, *The Wounded Storyteller*, xx, 53.

9 Frank, *The Wounded Storyteller*, xvii, xi.

10 Peterkin and Prettyman, ‘Finding a Voice’; Brown and Augusta-Scott, *Narrative Therapy*.

11 Shapiro, ‘The Role of Eye Movement Desensitization and Reprocessing (EMDR) Therapy in Medicine’.

12 Woods, ‘Post-Narrative — An Appeal’, 404.

13 Mantel, *Giving up the Ghost*; Kaysen, *The Camera My Mother Gave Me*. Subsequent references will be to these editions, and will be cited parenthetically in the text.

14 Susanna Kaysen, Author Q&A.

15 Bouson, *Embodied Shame*; Dolezal, *The Body and Shame*; Ahmed, *The Cultural Politics of Emotion*, 101–21; Kaufman, *The Psychology of Shame*; Pattison, *Shame*.

16 Morrison, *The Culture of Shame*, 33; Lynd, *On Shame and the Search for Identity*, 23–24.

17 Bartky, *Femininity and Domination*, 96.

18 See, for example: Dolezal and Lyons, ‘Health-Related Shame’; Dolezal, ‘The Horizons of Chronic Shame’; Hutchinson and Dhairyawan, ‘Shame, Stigma, HIV’; Loughlin et al., ‘Philosophy and the Clinic’; Cooper, Dolezal, and Rose, *COVID-19 and Shame*; Lyons and Dolezal, ‘Shame, Stigma and Medicine’; Dolezal, ‘The Phenomenology of Shame in the Clinical Encounter’.

19
**[Redacted for the purposes of this review]**

20 Lazare, ‘Shame and Humiliation in the Medical Encounter’; Nussbaum, *Hiding from Humanity Disgust, Shame, and the Law*, 174.

21 Mantel, ‘“How Much Pain Is Too Much Pain?”‘, 9.

22 Sörensdotter, ‘Pain and Sex(Uality) among Women Suffering from Vulvar Pain’, 64.

23 Bouson, *Embodied Shame*, 2–3.

24 Mitchell, *Writing Shame*, 16.

25 Frank, *Letting Stories Breathe*, 17.

26 Frank, *The Wounded Storyteller*, 5. Emphasis in original.

27 Frank, ‘Asking the Right Question about Pain’, 209.

28 For a discussion of shame and writing, see: Sheils and Walsh, *Shame and Modern Writing*; Mitchell, *Writing Shame*.

29 Pattison, *Shame*, 72.

30 Kirkpatrick, ‘MediaTalk; Setting New Frontiers in the Autobiography’.

31 Riley, ‘Lyric Shame’, 72.

32 Scarry, *The Body in Pain*.

33 Pattison, *Shame*, 71; Kaufman, *The Psychology of Shame*, 18–19.

34 Eakin, *Living Autobiographically*, 34.

35 Susanna Kaysen, Author Q&A; Blake, ‘P.S. Ideas, Interviews & Features…’, 7.

36 Frank, *The Wounded Storyteller*, 17.

37 Sedgwick, Frank, and Alexander, *Shame and Its Sisters*, 134–35.

38 Mitchell, *Writing Shame*, 28.

39 Class, ‘Reading the Assault on the Lived Body in Hilary Mantel’s Giving up the Ghost’; Vickers, ‘Illness and Femininity in Hilary Mantel’s *Giving Up the Ghost* (2003)’.

40 Blake, ‘P.S. Ideas, Interviews & Features…’, 7.

41 Lewis, ‘Shame and Stigma’, 127.

42 Ahmed, *The Cultural Politics of Emotion*, 105. Emphasis in original.

43 Nimnuan, Hotopf, and Wessely, ‘Medically Unexplained Symptoms’.

44 Blake, ‘P.S. Ideas, Interviews & Features…’, 7.

45 Susanna Kaysen, Author Q&A.

46 Woods, ‘Post-Narrative — An Appeal’, 404.

47 Bouson, *Embodied Shame*, 14.

48 Mitchell, *Writing Shame*, 26–27.

49 Bouson, *Embodied Shame*, 14.

50 Schaefert et al., ‘Non-Specific, Functional, and Somatoform Bodily Complaints’; Leiknes et al., ‘Course and Predictors of Medically Unexplained Pain Symptoms in the General Population’.
